# Treatment with intravenous immunoglobulins and methylprednisolone may significantly decrease loss of renal function in chronic-active antibody-mediated rejection

**DOI:** 10.1186/s12882-019-1385-z

**Published:** 2019-06-14

**Authors:** Kasia A. Sablik, Marian C. Clahsen-van Groningen, Caspar W. N. Looman, Jeffrey Damman, Madelon van Agteren, Michiel G. H. Betjes

**Affiliations:** 1 Erasmus Medical Center, Department of Nephrology & Transplantation, room Na2105, P.O. Box 2040, 3000 CA Rotterdam, The Netherlands; 2 Department of Pathology, Erasmus Medical Center, Rotterdam, The Netherlands; 3 Department of Biostatistics, Erasmus Medical Center, Rotterdam, The Netherlands

**Keywords:** Transplantation, Renal allograft rejection, C-aABMR, IVIG, MP, Treatment

## Abstract

**Background:**

Chronic-active antibody mediated rejection (c-aABMR) is a major contributor to long-term kidney allograft loss. We conducted a retrospective analysis to establish the efficacy of treatment with intravenous immunoglobulins (IVIG) and pulse methylprednisolone (MP) of patients with c-aABMR.

**Methods:**

Sixty-nine patients, in the period 2005–2017, with the diagnosis (suspicious for) c-aABMR that were treated with IVIG and MP were included. Patients were administered three doses of 1 g intravenous MP combined with a single dose of IVIG (1 g/kg body weight). Primary outcome was the decline in allograft function one year post treatment. Responders to IVIG-MP therapy were defined by an eGFR one year after treatment which was at least 25% above the projected allograft function.

**Results:**

Patients showed an average decline in eGFR of 9.8 ml/min/1.73m^2^ the year prior to treatment. Following treatment, a significant reduction (*p* < 0.001) in eGFR decline was observed (6.3 ml/min/1.73m^2^). Furthermore, a significant improvement in proteinuria was observed upon treatment (*p* < 0.001). Sixty-two percent (*n* = 43) of the patients were considered a responder and showed considerable slowing of graft function deterioration in the year after treatment (*p* < 0.001). Three and 5-year graft survival was significantly superior in responders.

**Conclusions:**

More than 60% of patients with c-aABMR with a progressive decline in eGFR respond favorably to treatment with IVIG-MP resulting in a significant improvement of graft survival (Sablik, Am J Transplant 18, 2018).

**Electronic supplementary material:**

The online version of this article (10.1186/s12882-019-1385-z) contains supplementary material, which is available to authorized users.

## Background

Short-term outcome of kidney transplants has improved significantly due to the introduction of calcineurin inhibitors (CNI), induction therapy with T cell depleting agents and IL-2 receptor blocker [1–3]. However, improvement in long-term renal allograft survival still presents a considerable clinical problem [4–7]. In recent years, chronic-active antibody mediated rejection (c-aABMR) has become recognized as one of the major barriers for long term renal allograft survival [7–9].

Advanced c-aABMR often presents itself as a progressive loss of allograft function, in addition to progressive proteinuria and hypertension. Renal allograft survival is poor as most patients develop allograft failure within 2 years after being diagnosed with c-aABMR [7, 10–13]. It is therefore important to find therapeutic options for c-aABMR that are aimed at stabilizing or slowing the decrease in allograft function.

Currently, only little is published about the efficacy of treatment after c-aABMR has been diagnosed. A number of studies have indicated that the use of rituximab (RTX), tocilizumab, bortezomib, intravenous immunoglobulins (IVIG) therapy and/or plasmapheresis (PP), may favorably attenuate the loss of allograft function in patients with chronic ABMR with or without transplant glomerulopathy (TG) [14–19]. However, these studies were uncontrolled and conducted with small numbers of patients over relatively short periods of time. The recently published, first and only randomized, placebo-controlled trial in late ABMR (BORTEJECT), showed disappointing results upon treatment with bortezomib as no improvement in eGFR loss was achieved [20].

Our renal transplant center has, in the last decade, adopted the policy to treat patients with c-aABMR with a single course of IVIG and pulse intravenous MP based on favorable initial results. In this study we retrospectively analyzed the efficacy of this therapy in a group of c-aABMR patients.

## Methods

### Study population

**Fig. 1:**
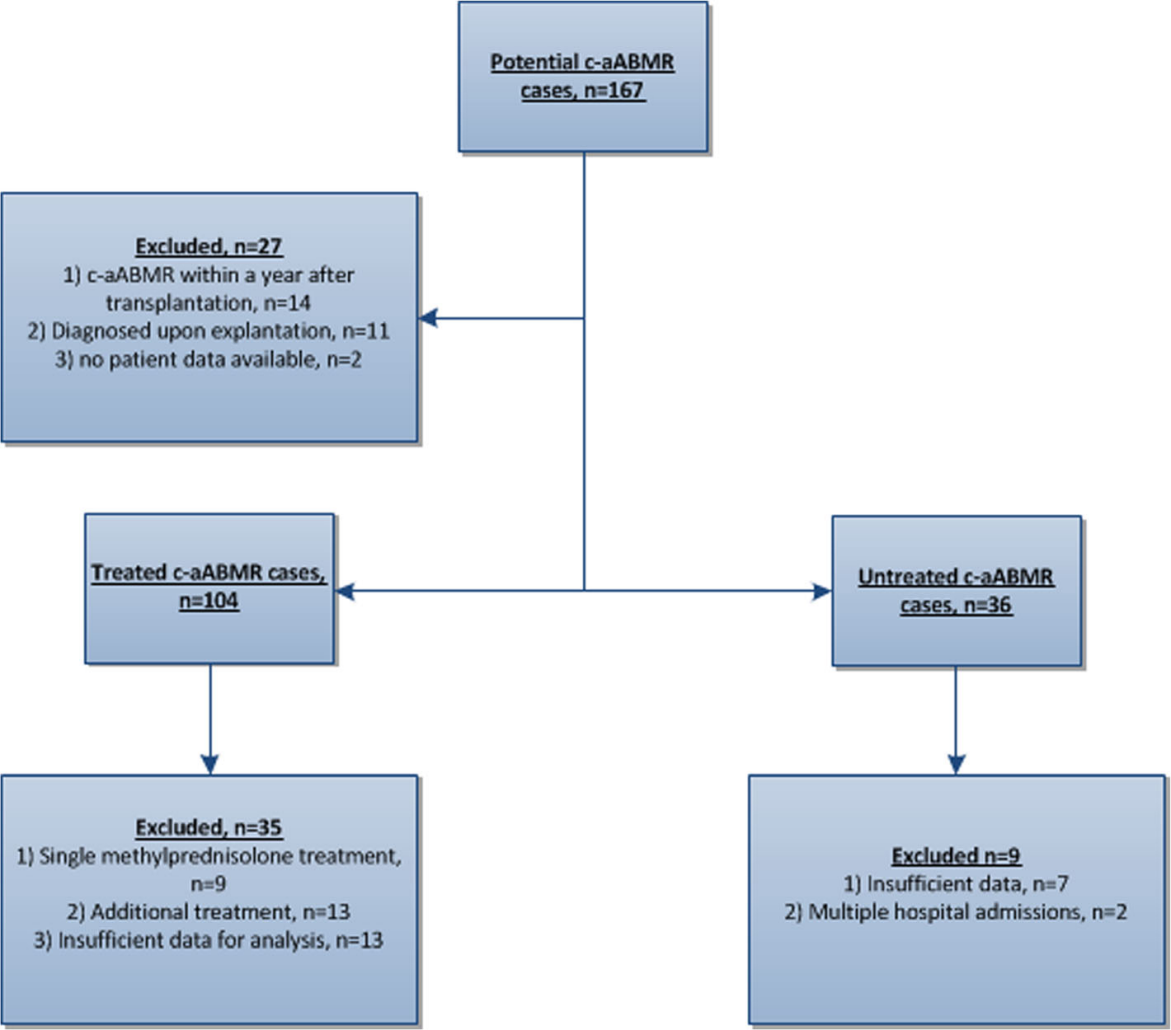
Patient selection flow chart

We retrospectively identified renal transplant recipients with biopsy proven (suspicious for) c-aABMR at the Erasmus Medical Center between January 2005 and January 2017. Patients were identified from the pathology database at our center. A total of 167 patients were found eligible for inclusion (Fig. 1). Patients with c-aABMR diagnosed at least one year after transplantation were eligible for evaluation of the effect of IVIG-MP treatment on the progressive decrease in graft function. The inclusion criteria were treatment with three doses of 1 g intravenous MP over a 3 day period combined with a single dose of IVIG (1 g/kg body weight) and sufficient data (see below) on allograft function for analysis and if no additional treatment was given (e.g. Alemtuzumab, Anti-thymocyte globulin). Sixty-nine patients were found eligible and were included as cases. Additionally, a historical patient group (*n* = 27) was identified that did not receive any additional treatment upon c-aABMR diagnosis. Most of these patients were diagnosed with c-aABMR before adaptation of the local treatment guideline for c-aABMR in 2008.

All renal biopsies were for cause and evaluated at time of biopsy by an experienced renal pathologist based on the then current Banff classification [21–25]. Alternative diagnoses for the histomorphological changes compatible with c-aABMR such as membranoproliferative glomerulonephritis (MPGN) or (chronic) thrombotic microangiopathy (cTMA) were excluded, either by immunofluorescence or clinical analysis.

This retrospective study was reviewed and approved by the Institutional Ethics Committee from the Erasmus MC, Rotterdam, The Netherlands. As this was a retrospective study, written consent to participate from the study subjects was not required.

### Data collection

Demographic and baseline transplantation characteristics were collected for all patients, as well as data on renal allograft function and proteinuria. Renal allograft function was assessed on the basis of estimated glomerular filtration rate (eGFR) by means of modification of diet in renal disease (MDRD) [26]. All eGFR measurements sampled during outpatient clinic visits in the year prior to and year post diagnosis and/or treatment with IVIG-MP were included. Measurements taken during hospitalization were omitted from analysis to minimize bias due to, for example, the admission of intravenous fluids, infection, etc. Patients needed at least 7 measurements of eGFR at regular intervals in the year before and after treatment with IVIG-MP. This was considered the minimal number of data to perform multilevel statistical analysis (see below). Allograft failure was defined as the need for dialysis or kidney re-transplantation.

Similarly, data on proteinuria was collected also with a minimum of 7 measurements at regular intervals in the year before and after treatment with IVIG-MP.

### Statistical analysis

Normally distributed data are expressed as mean +/− SD, non-normally distributed data as median (IQR/range). A *P*-value of less than 0.05 was considered statistically significant. All statistical analysis were performed using the R statistical programming environment and SPSS software version 21.

The primary outcome of this study was the efficacy of IVIG-MP therapy on allograft function in the first year after treatment. This was analyzed by 2 different strategies. First, the treatment efficacy was explored by means of multilevel analysis. This model allows for the detection of a change in allograft function based on the statistical analysis of longitudinal data with intercept, slope and number of observations varying across all patients. It is considered a successful method for providing a reliable estimate of change in allograft function over time [27]. This type of analysis shows the statistical significance of the impact of IVIG-MP treatment for the whole group of patients.

Secondly, all cases were classified as either responder or non-responder. In order to stratify patients we determined the slope of allograft deterioration for each individual patient. Based on their slope 1 year prior to diagnosis, a projection was made for their predicted graft function one year after diagnosis. Patients with a measured eGFR at one year after treatment of at least 25% above the projected eGFR were classified as responder. To verify whether this stratification is warranted, additional multilevel analysis was performed for the responders as a group and the non-responders as a group. Graft survival censored for death after IVIG-MP treatment was calculated using Kaplan-Meier curves and log rank statistics. The efficacy of the IVIG-MP treatment on proteinuria was analyzed similarly. The clinical and demographical characteristics of responders and non-responders were compared using unpaired t test for continuous variables, Mann Whitney U test for ordinal variables and Chi-squared or Fisher exact test for categorical variables.

## Results

### Patient characteristics

The demographic and clinical characteristics for the 69 cases at time of for-cause biopsy are summarized in Table 1 (Additional file 1: Table S1, data on historic controls). On average patients were 53 years old and a majority was transplanted with a living donor kidney (75%). The maintenance immunosuppressive regimen predominantly consisted of double immunosuppression with a combination of tacrolimus and mycophenolate mofetil. Steroids were used in less than half of the patients (46%).

**Table 1:** Clinical and demographic characteristics (record at time of c-aABMR diagnosis)

	Total (*n* = 69)	Responders (n = 43)	Non-Responders (n = 26)	p-value
Women, n (%)	25 (36)	14 (48)	11 (42)	0.41
Age of patient, yr, median (IQR)	53 (42–66)	54 (42–66)	52 (42–62)	0.40
Living donor, n (%)	52 (75)	35 (81)	17 (65)	0.14
Prior kidney transplant, n (%)	17 (25)	10 (23)	7 (27)	0.73
Donor age, yr, median (IQR)	50 (41–57)	53 (43–61)	47 (40–52)	0.05
PRA current, median (IQR)	0 (0–4)	0 (0–4)	0 (0–5)	0.79
HLA mismatch, median (IQR)	3 (2–4)	3 (2–4)	3 (3–4)	0.24
Maintenance immunosuppression, n (%)				>0.05
❖ Tacrolimus/cyclosporine	57 (83)	37 (86)	20 (77)	
❖ mTOR inhibitor	6 (9)	3 (7)	3 (12)	
❖ Steroids	32 (46)	22 (51)	10 (38)	
❖ Mycophenolate mofetil	59 (86)	37 (86)	22 (85)	
❖ Other	2 (3)	0 (0)	2 (8)	
Maintenance immunosuppression, n (%)				0.58
❖ Triple immunosuppression	21 (30)	15 (35)	6 (23)	
❖ Double immunosuppression	44 (64)	25 (58)	19 (73)	
❖ Single immunosuppression	4 (6)	3 (7)	1 (4)	
Primary kidney disease, n (%)				0.65
❖ Diabetic nephropathy	7 (10)	5 (12)	2 (7)	
❖ Hypertensive nephropathy	9 (13)	5 (12)	4 (15)	
❖ Polycystic kidney disease	8 (12)	4 (9)	4 (15)	
❖ Primary glomerulopathy	19 (28)	12 (28)	7 (27)	
❖ Reflux nephropathy	5 (7)	2 (5)	3 (12)	
❖ Chronic pyelonephritis	3 (4)	1 (2)	2 (7)	
❖ Other	15 (22)	12 (28)	3 (12)	
❖ Unknown	3 (4)	2 (5)	1 (4)	
Time to c-aABMR, yr, median (IQR)	6.3 (2.8–9.2)	6.1 (2.7–8.9)	6.5 (3.6–10.5)	0.32
eGFR (ml/min/1.73m^2^), mean (SD)	34 (±2.0)	33 (±2.5)	36 (±3.4)	0.15
eGFR measurements, n, median (IQR)	19 (14–24)	18 (13–22)	19 (15–26)	0.51
Proteinuria (mg/mmol), mean (SD)	230 (157–302)	219 (108–329)	250 (178–323)	0.69

Time to biopsy from the moment of transplantation showed a median of 6.3 years (2.8–9.2 years). Patients had a median eGFR of 34 mL/min/1.73m^2^ and 230 mg/mmol proteinuria at time of renal biopsy. No deaths occurred within the first year of follow-up.

### Renal allograft function and response to IVIG-MP treatment

**Fig. 2:**
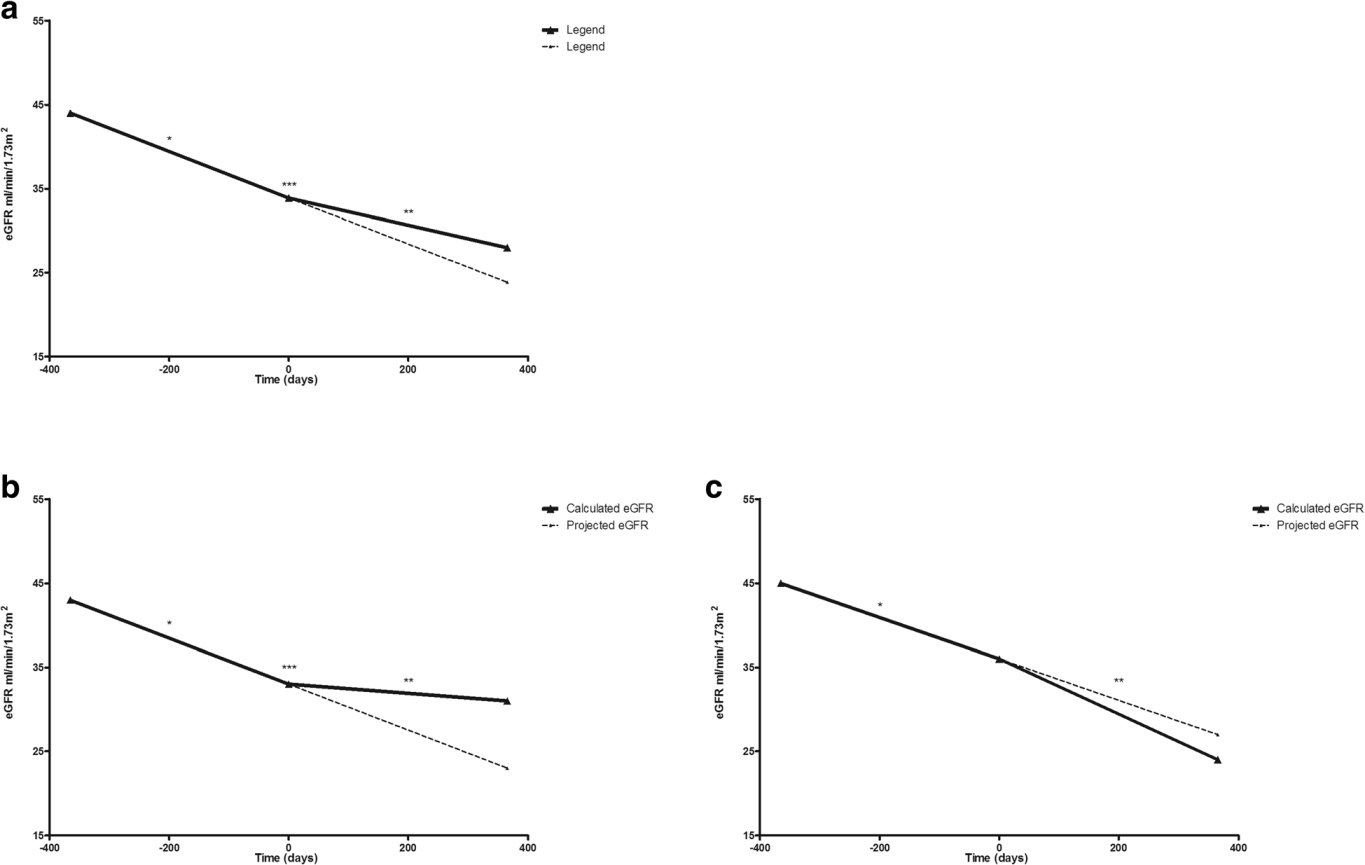
**a)** Renal allograft function and response to therapy. The renal allograft function of the 69 patients one year before and one year after IVIG-MP treatment (t_0_). *Calculated average decline in eGFR of 9.8 ml/min/1.73m^2^ (p < 0.001) **Calculated average decline in eGFR of 6.3 ml/min/1.73m^2^ (p < 0.001) ***change in slope of renal allograft function in the year before and after treatment (*p* < 0.001). **b)** Renal allograft function and response to therapy of the responders. The renal allograft function of the 43 cases one year before and one year after IVIG-MP treatment (t _0_). *Calculated average decline in eGFR of 10.3 ml/min/1.73m^2^ (p < 0.001), **Calculated average decline in eGFR of 2.0 ml/min/1.73m^2^ (p < 0.001), ***change in renal allograft function (p < 0.001). **c)** Renal allograft function and response to therapy of non-responders. The renal allograft function of the 26 patients one year before and one year after IVIG-MP treatment (t_0_). *Calculated average decline in eGFR of 9.1 ml/min/1.73m^2^ (p < 0.001) **Calculated average decline in eGFR of 12.0 ml/min/1.73m^2^ (p < 0.001)

Renal allograft function of the 69 patients treated with IVIG-MP declined from an average eGFR of 44 ml/min/1.73m^2^ at 1 year prior to therapy to 34 ml/min/1.73m^2^ at time of treatment (t0, IVIG-MP treatment). The year after treatment, allograft function further declined to 28 ml/min/1.73m^2^. The course of eGFR is illustrated in Fig. 2a. The calculated average decline in eGFR of 6.3 (5.3–7.3) ml/min/1.73m^2^ in the year after IVIG-MP treatment was significantly less than the average decline of 9.8 (8.9–10.8) ml/min/1.73m^2^ in the year prior to treatment (multilevel analysis, *p* < 0.001).

**Fig. 3:**
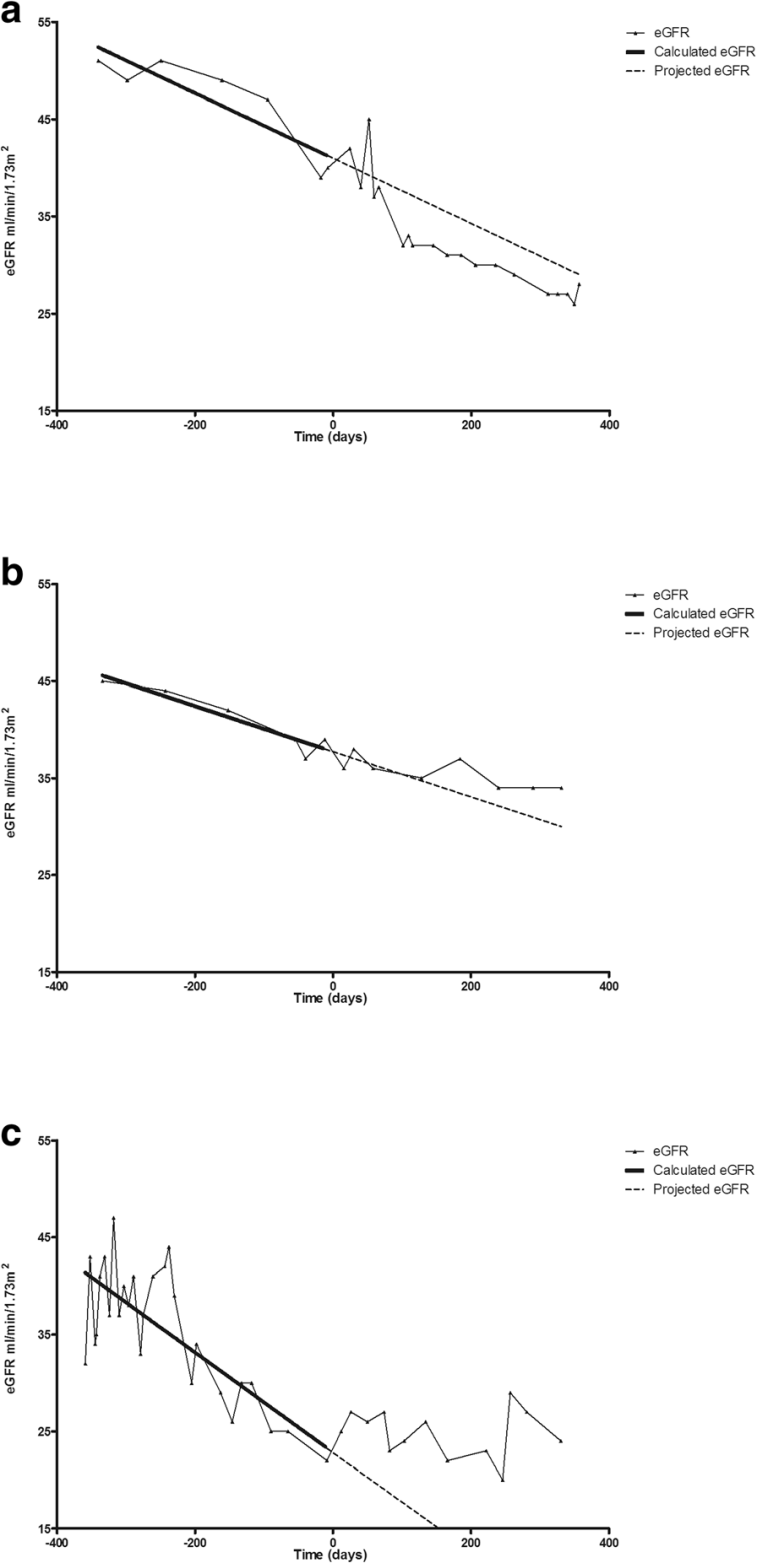
Patterns of response to treatment with IVIG-MP for c-aABMR related progressive loss of renal function. **a)** Typical example of a non-responder; **b)** typical example of a responder with significant slowing of the progressive decline in eGFR; **c)** typical example of a responder with stabilization of the renal allograft function after treatment

Sixty-two percent of patients (*n* = 43) had an eGFR one year after treatment of at least 25% above the projected eGFR and were categorized as responders. Figure 3 shows typical examples of a non-responder (Fig. 3a), a responder showing a significant slowing of progressive eGFR loss (Fig. 3b) and a responder with a stabilization of renal function after treatment (Fig. 3c). The latter 2 patterns of response were most frequent as improvement of eGFR within the year after treatment was usually not observed.

As a group, the responders had a decline in allograft function from an eGFR of 43 ml/min/1.73m^2^ 1 year prior to treatment to 33 ml/min/1.73m^2^ at time of treatment (decline in eGFR; 10.3 (9.2–11.5) ml/min/1.73m^2^). The calculated average decline in eGFR after IVIG-MP treatment was 2.0 (0.9–3.4) ml/min/1.73m^2^ (Fig. 2b). The non-responders (*n* = 26) showed an average decline in eGFR from 45 ml/min/1.73m^2^ 1 year prior to treatment to 36 ml/min/1.73m^2^ at time of treatment (decline in eGFR; 9.1 (7.6–10.6) ml/min/1.73m^2^) which was similar to the group of responders. The allograft function in the year prior to treatment did not differ between responders and non-responders (*p* = 0.19). In non-responders eGFR declined to 24 ml/min/1.73m^2^ 1 year after treatment (Fig. 2c; decline in eGFR 12.0 (10.6–13.5) ml/min/1.73m^2^).

Similar to the treated patients, eGFR loss was calculated in the untreated historic patient group (*n* = 27) before and after the diagnosis. The average decline in eGFR was 11.3 (9.5–13.0) ml/min/1.73m^2^ and 9.7 (7.9–11.6) ml/min/1.73m^2^ respectively, with no significant change in graft function (*p* = 0.33). These values are in range with the decline in eGFR prior to diagnosis in patients treated with IVIG-MP and in accordance with the unfavorable prognosis of c-aABMR as described in literature [12].

### Renal allograft survival after IVIG-MP treatment

**Fig. 4:**
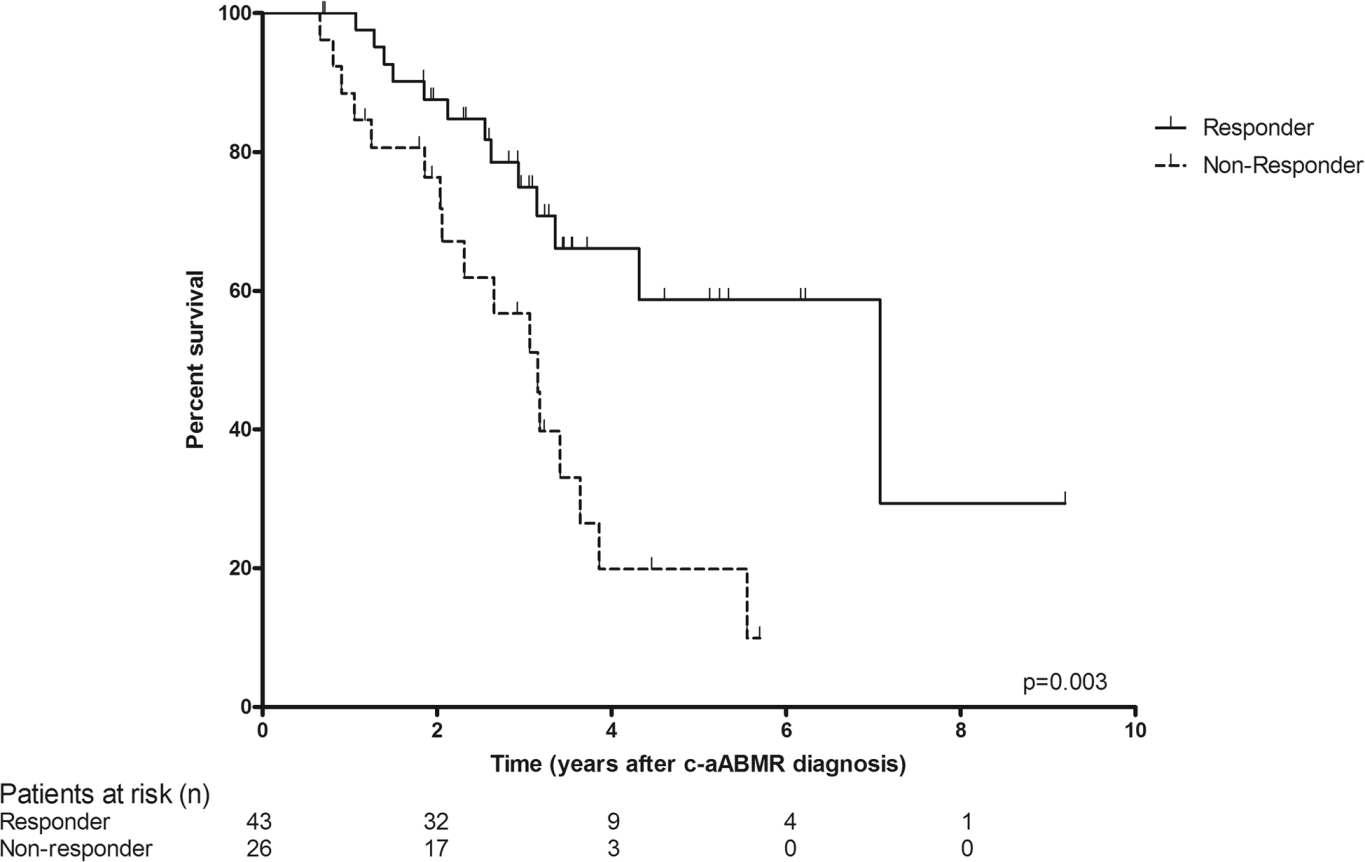
Kaplan-Meier curves for renal graft survival censored for death for responders (n = 43) versus non-responders (n = 26) (log rank; p = 0.003)

Three patients (non-responders) out of the 69 treated patients showed relentless progression of disease to graft failure and returned to dialysis within the first year after IVIG-MP administration. The graft survival censored for death after c-aABMR diagnosis for the responders and non-responders is shown in Fig. 4. The responders had a significantly improved graft survival with a mean survival of 5.9 (4.5–7.2) year versus 3.1 (2.4–3.8) year for the non-responders (*p* = 0.003). Graft survival was similar for non-responders and the historic untreated patient group (*p* = 0.94).

Graft survival after c-aABMR diagnosis at 1, 3 and 5 years was 100, 75 and 59%, respectively in the group of responders versus 89, 57 and 20% for the non-responders. The group of untreated patients had similar survival to the non-responders with 74, 38 and 33%, respectively.

### Difference between responders and non-responders in relation to clinical variables

Of all clinical variables, only age of the donor was found to be significantly different between responders and non-responders (Table 1). On average responders had a slightly older donor than non-responders (53 vs. 47 years; *p* = 0.046). However, responders more often received a kidney from a living donor although this was not statistically significant (81% vs. 65%, *p* = 0.14). There was no difference in HLA mismatch nor whether the patient has received a previous kidney transplant.

Renal allograft function at time of c-aABMR diagnosis was similar as was proteinuria between the 2 patient groups (*p* = 0.15; *p* = 0.69). The 2 groups received comparable immunosuppressive therapy, both in number and type of treatment. Over 85% of patients received mycophenolate mofetil as maintenance immunosuppression in addition to a calcineurin inhibitor in over 80% of patients.

### Proteinuria and response to IVIG-MP treatment

**Fig. 5:**
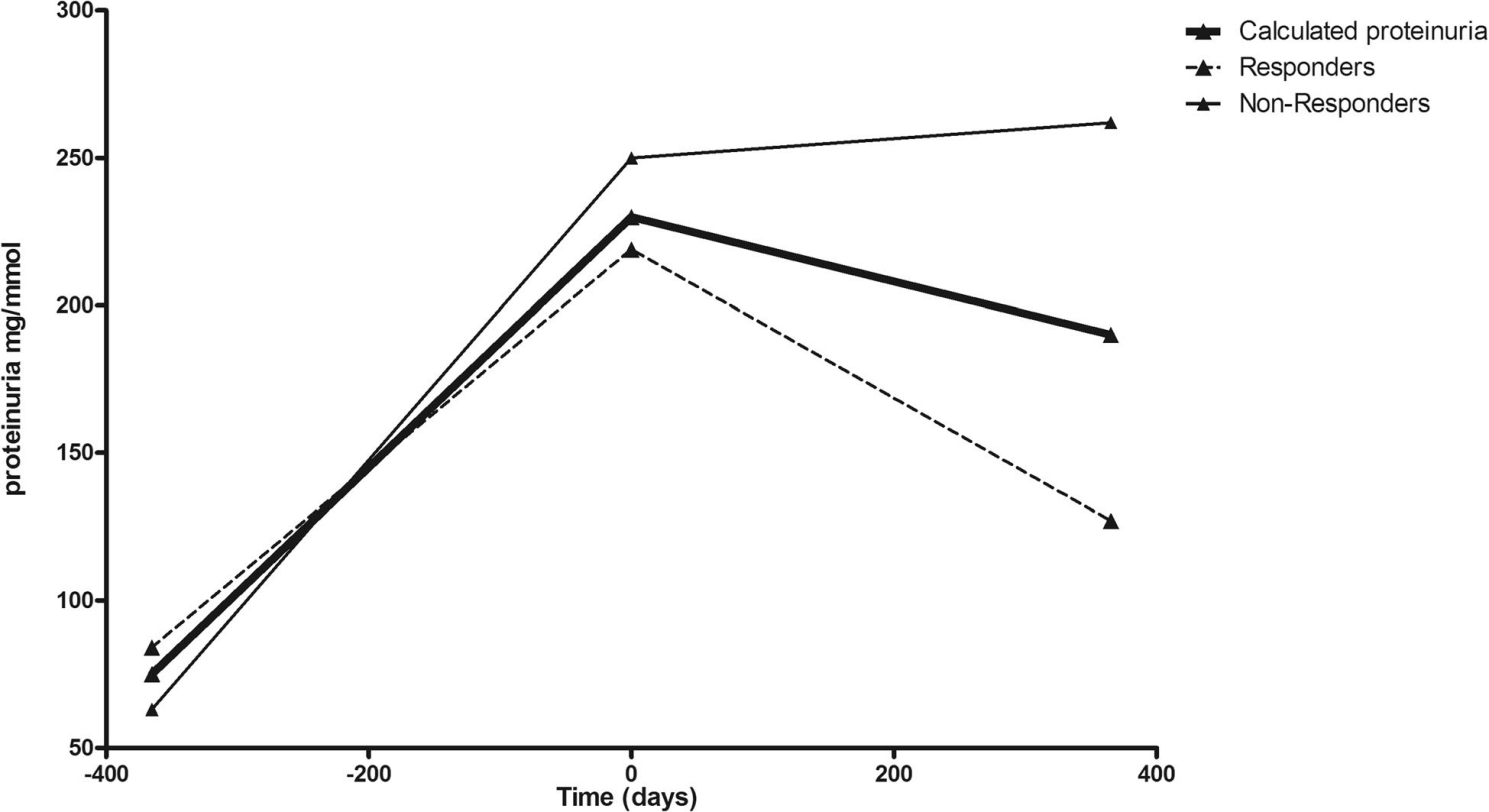
Proteinuria one year before and one year after IVIG-MP treatment (t_0_). Calculated average proteinuria of the whole group of treated patients (*n* = 41; bold line) and both responders (*n* = 25; continuous line) and non-responders (*n* = 16; dotted line)

Effect of treatment on proteinuria was analyzed and compared for 41 patients. Sufficient data for statistical analysis was missing for the remaining 28 patients. On average IVIG-MP therapy was associated with a decrease in proteinuria level the first year after administration. Proteinuria initially increased in the 41 patients from 75 mg/mmol 1 year before therapy to 229 mg/mmol at time of treatment (Fig. 5). In the year after treatment with IVIG-MP, proteinuria declined from 229 mg/mmol to 190 mg/mmol. The calculated average decline in proteinuria of 39 (1–79) mg/mmol the first year after IVIG-MP treatment differed significantly (*p* < 0.001) from the average increase in proteinuria of 154 (117–192) mg/mmol in the year before treatment. Of the 41 treated patients, 25 were previously classified as responders and 16 as non-responders. Although both groups showed a significant response to treatment (< 0.001), the non-responders showed a near stabilization of proteinuria, whereas the responders had a decrease of 92 mg/mmol in the year after treatment (Fig. 5).

At time of treatment, 44% (*n* = 18) of patients were on anti-proteinuric treatment with either an ACE inhibitor and/or ARBs. Anti-proteinuric treatment was initiated in 15% of patients (*n* = 6) shortly after IVIG-MP treatment and treatment was stopped for 1 patients. The remaining patients had no additional form of anti-proteinuric treatment (39%, *n* = 16).

## Discussion

Chronic-active Antibody Mediated Rejection (c-aABMR) is an important cause of renal allograft dysfunction over time, often presenting as a continuous decline in allograft function, either with or without proteinuria [7, 9–13]. Despite the growing awareness of the clinical significance, there is no consensus on an effective therapeutic regimen for patients with c-aABMR [28, 29]. Our data show that IVIG-MP treatment of patients with c-aABMR has the potential to significantly slow the progression of eGFR loss within the first year after IVIG-MP administration.

There have been several studies published on the therapeutic approach of late or chronic ABMR. The majority of these case-series report about their experience with known desensitization protocols in various combinations (IVIG, plasmapheresis, rituximab, tocilizumab) [16, 18, 19, 30–32]. Although the results are inconsistent, several studies have shown a positive effect of therapy within the first year after treatment which translated in improved allograft survival. Other studies have shown that treatment (IVIG-rituximab) for chronic ABMR does not seem to alter the progression of decrease in allograft function [19].

Problematic in the afore mentioned studies are the relatively small number of cases, the heterogeneity in additional immune suppressive treatment and often the lack of thorough statistical analysis of loss of allograft function over time. Only recently the first randomized, placebo-controlled trial for late ABMR was published using bortezomib in the treatment arm. Unfortunately, allograft function did not significantly differ between the control and treatment arm [20].

In the current study, we analyzed a group of patients with c-aABMR. Overall, there was a significant decrease in eGFR loss when the decline in graft function in the year before treatment was compared to the decrease in graft function the year after treatment. However, it was clear that the response to treatment was heterogeneous while in about 2/3 of treated patients a significant treatment response was achieved. The remaining patients had a relatively unchanged loss of eGFR after treatment. The treatment effect in the responders translated in improved graft survival which was nearly double of that of the non-responders. Of interest is that both allograft function and survival were similar in the non-responders and the historic untreated group of c-aABMR patients.

This study has some obvious limitations. The study is retrospective in nature allowing for unknown bias due to a selection of patients with a for-cause biopsy. It also excludes the possibility of a uniform schedule of, for example, maintenance immunosuppression. It does however, allow for a proper analyses of the decline in allograft function. The multivariate analysis allows each treated patient to be its own control comparing the eGFR loss before and after treatment, strengthening our finding that IVIG-MP seems to be beneficial in diminishing eGFR loss caused by c-aABMR. And although the studied population is relatively small, it is to date, one of the larger patient groups who have had a similar treatment regimen after the diagnosis of c-aABMR. In addition, significant response to treatment was unmistakable in some patients as shown in the figures.

## Conclusion

In conclusion, immunosuppressive therapy with IVIG-MP may significantly slow eGFR loss in a substantial proportion of patients with c-aABMR leading to improved graft survival. As c-aABMR is now recognized as a major cause of long term renal allograft loss, the efficacy of IVIG-MP treatment is an important and hopeful finding.

## Additional file

Additional file 1: **Table S1**. Clinical and demographic characteristics of cases and historic controls. (DOCX 16 kb)
