# Supplementary material for: Treatment with intravenous immunoglobulins and methylprednisolone may significantly decrease loss of renal function in chronic-active antibody-mediated rejection
Source: BMC Nephrol. 2019 Jun 14;20:218. doi: 10.1186/s12882-019-1385-z (PMC6567552; doi:10.1186/s12882-019-1385-z)
Supplement: Supplementary file 1 — Table S1. Clinical and demographic characteristics of cases and historic controls. (DOCX 16 kb) [file 12882_2019_1385_MOESM1_ESM.docx]

**Supplementary Table 1.** Clinical and demographic characteristics of cases and historic controls.

|  | **Total (n=69)** | **Historic control (n=27)** | **p-value** |
| --- | --- | --- | --- |
| **Women, n (%)** | 25 (36) | 16 (59) | 0.04 |
| **Age of patient, yr, median (IQR)** | 53 (42-66) | 51 (28-58) | 0.06 |
| **Living donor, n (%)** | 52 (75) | 16 (64) | 0.28 |
| **Prior kidney transplant, n (%)** | 17 (25) | 9 (33) | 0.28 |
| **Donor age, yr, median (IQR)** | 50 (41-57) | 49 (40-54) | 0.75 |
| **PRA current, median (IQR)** | 0 (0-4) | 4 (0-44) | 0.06 |
| **HLA mismatch, median (IQR)** | 3 (2-4) | 3 (2-4) | 0.20 |
| **Maintenance immunosuppression, n (%)**   - **Tacrolimus/cyclosporine** - **mTOR inhibitor** - **Steroids** - **Mycophenolate mofetil** - **Other** | 57 (83)  6 (9)  32 (46)  59 (86)  2 (3) | 15 (63)  2 (8)  15 (63)  18 (69)  2 (8) | >0.05 |
| **Maintenance immunosuppression, n (%)**   - **Triple immunosuppression** - **Double immunosuppression** - **Single immunosuppression** | 21 (30)  44 (64)  4 (6) | 4 (15)  20 (75)  0 (0) | 0.16 |
| **Primary kidney disease, n (%)**   - **Diabetic nephropathy** - **Hypertensive nephropathy** - **Polycystic kidney disease** - **Primary glomerulopathy** - **Reflux nephropathy** - **Chronic pyelonephritis** - **Other** - **Unknown** | 7 (10)  9 (13)  8 (12)  19 (28)  5 (7)  3 (4)  15 (22)  3 (4) | 1 (4)  4 (15)  4 (15)  6 (22)  1 (4)  1 (4)  5 (18)  5 (18) | 0.48 |
| **Time to c-aABMR, yr, median (IQR)** | 6.3 (2.8-9.2) | 5.5 (2.5-8.9) | 0.82 |
| **eGFR (ml/min/1.73m^2^), mean (SD)** | 34 (±2.0) | 31(±2.0) | 0.37 |
| **eGFR measurements, n, median (IQR)** | 19 (14-24) | 17 (13-21) | 0.67 |
| **Proteinuria (mg/mmol), mean (SD)** | 230 (157-302) | - | - |
